# A 13-gene expression-based radioresistance score highlights the heterogeneity in the response to radiation therapy across HPV-negative HNSCC molecular subtypes

**DOI:** 10.1186/s12916-017-0929-y

**Published:** 2017-09-01

**Authors:** Jean-Philippe Foy, Louis Bazire, Sandra Ortiz-Cuaran, Sophie Deneuve, Janice Kielbassa, Emilie Thomas, Alain Viari, Alain Puisieux, Patrick Goudot, Chloé Bertolus, Nicolas Foray, Youlia Kirova, Pierre Verrelle, Pierre Saintigny

**Affiliations:** 1 Univ Lyon, Université Claude Bernard Lyon 1, INSERM 1052, CNRS 5286, Centre Léon Bérard, Centre de recherche en cancérologie de Lyon, Lyon, F-69008 France; 2 Department of Translational Research and Innovation, Centre Léon Bérard, Lyon, F-69008 France; 3 Department of Oral and Maxillofacial Surgery, University of Pierre Marie Curie-Paris 6, Pitié-Salpêtrière Hospital, Paris, F-75013 France; 4 Department of Radiation Oncology, Institut Curie, Paris, F-75005 France; 5 Department of Surgery, Centre Léon Bérard, Lyon, F-69008 France; 6 Platform of Bioninformatics-Gilles Thomas, Synergie Lyon Cancer, Lyon, F-69008 France; 7 INSERM U 1196 , CNRS UMR 9187, Institut Curie, Orsay, F-91405 France; 8 Université Clermont Auvergne, Centre Jean-Perrin, Clermont-Ferrand, F-63000 France; 9 Department of Medical Oncology, Centre Léon Bérard, Lyon, 69008 France

**Keywords:** Head neck squamous cell carcinomas, Molecular subtypes, Predictive biomarker, Radiation therapy, Relapse, Resistance

## Abstract

**Background:**

Radiotherapy for head and neck squamous cell carcinomas (HNSCC) is associated with a substantial morbidity and inconsistent efficacy. Human papillomavirus (HPV)-positive status is recognized as a marker of increased radiosensitivity. Our goal was to identify molecular markers associated with benefit to radiotherapy in patients with HPV-negative disease.

**Methods:**

Gene expression profiles from public repositories were downloaded for data mining. Training sets included 421 HPV-negative HNSCC tumors from The Cancer Genome Atlas (TCGA) and 32 HNSCC cell lines with available radiosensitivity data (GSE79368). A radioresistance (RadR) score was computed using the single sample Gene Set Enrichment Analysis tool. The validation sets included two panels of cell lines (NCI-60 and GSE21644) and HPV-negative HNSCC tumor datasets, including 44 (GSE6631), 82 (GSE39366), and 179 (GSE65858) patients, respectively. We finally performed an integrated analysis of the RadR score with known recurrent genomic alterations in HNSCC, patterns of protein expression, biological hallmarks, and patterns of drug sensitivity using TCGA and the E-MTAB-3610 dataset (659 pancancer cell lines, 140 drugs).

**Results:**

We identified 13 genes differentially expressed between tumor and normal head and neck mucosa that were associated with radioresistance in vitro and in patients. The 13-gene expression-based RadR score was associated with recurrence in patients treated with surgery and adjuvant radiotherapy but not with surgery alone. It was significantly different among different molecular subtypes of HPV-negative HNSCC and was significantly lower in the “atypical” molecular subtype. An integrated analysis of RadR score with genomic alterations, protein expression, biological hallmarks and patterns of drug sensitivity showed a significant association with *CCND1* amplification, fibronectin expression, seven hallmarks (including epithelial-to-mesenchymal transition and unfolded protein response), and increased sensitivity to elesclomol, an HSP90 inhibitor.

**Conclusions:**

Our study highlights the clinical relevance of the molecular classification of HNSCC and the RadR score to refine radiation strategies in HPV-negative disease.

**Electronic supplementary material:**

The online version of this article (doi:10.1186/s12916-017-0929-y) contains supplementary material, which is available to authorized users.

## Background

Head and neck squamous cell carcinoma (HNSCC) is the sixth most common cancer worldwide and the second smoking-related cancer after lung carcinoma [1]. Management of HNSCC is challenging due to the high morbidity and mortality of surgery and radiotherapy [2]. Although intensity-modulated radiotherapy has greatly improved the quality of life of patients [3], especially by decreasing the incidence of early acute and late grade xerostomia [4], the identification of biomarkers that may help tailor radiotherapy is needed.

Several approaches have been used to predict the tumor response to radiotherapy, including functional assays (clonogenic cell survival [5] or DNA repair [6]) or omics-based biomarkers [3, 7–10]. The latter have been identified in cancer cell lines and their expression in normal cells has not been evaluated. Tumor samples from patients harbor a diverse range of stromal/normal cells that alter the tumor purity, which is known to influence the biological interpretation of results generated from genomic approaches [11–13]. Therefore, the potential impact of normal tissue contaminating the tumor sample on intrinsic radioresistance has to be considered.

Oropharyngeal human papillomavirus (HPV)-positive HNSCC are associated with an increased radiosensitivity and a favorable outcome compared to HPV-negative HNSCC [14–16]. De-escalation strategies are currently being evaluated in clinical trials in this setting [17, 18]. The higher radiosensitivity of HPV-positive HNSCC may be due to a more effective cytotoxic T-cell-related antitumor immune response [19], as well as specific molecular alterations, such as p16 overexpression, that decrease DNA repair by inhibiting the recruitment of RAD51 to the site of DNA damage [20]. In HPV-negative HNSCC, the epithelial-to-mesenchymal transition (EMT) via overexpression of fibronectin 1 (FN1), as well as activation of the epidermal growth factor receptor (*EGFR*) have been implicated in HNSCC radioresistance [9, 21–23]. Genome-wide expression profiling of a large number of HNSCC led to the identification of four robust molecular classes of HNSCC [24–26]. In this classification, the “classical”, “basal” and “mesenchymal” subtypes exhibit canonical genomic alterations such as focal *EGFR* amplification, high frequency of *HRAS* mutations, and upregulation of EMT-related genes, respectively [25, 26]. Therefore, it is tempting to hypothesize that these HNSCC classes are more radioresistant compared to the “atypical” class characterized by the lack of *EGFR* amplification and enriched in HPV-positive tumors. Of note, a significant proportion of HNSCC in the “atypical” subtype are HPV-negative and the radiosensitivity of these tumors is unknown.

In this work, we made use of gene expression profiles available in public repositories to define a 13-gene expression-based radioresistance (RadR) score in HPV-negative HNSCC and evaluate its association with the current molecular classification, known recurrent genomic alterations in HNSCC, protein expression, biological hallmarks, and patterns of drug sensitivity in vitro. We found the RadR score to be (1) associated with poor disease free-survival (DFS) in HPV-negative HNSCC patients treated by surgery and radiotherapy (with or without chemotherapy) but not in patients treated with surgery alone; (2) lower in the HPV-negative atypical molecular subtype and higher in the mesenchymal subtype; and (3) significantly associated with *CCND1* amplification, fibronectin expression and seven biological hallmarks, including the ENT and the actionable “unfolded protein response” hallmarks. Current molecular classification of HNSCC and the RadR score may help to tailor radiotherapy in HPV-negative HNSCC.

## Methods

No ethical approval was needed for this study.

### Cancer cell line datasets

We identified genome-wide expression profiles from four sets of established cancer cell lines with available radiation or drug sensitivity data. The first set included 59 cell lines established from 9 different cancer tissue types (breast, central nervous system, colon, leukemia, melanoma, non-small cell lung, ovarian, prostate, and renal) and included in the NCI-60 panel. Raw data was downloaded from the CellMiner database [27, 28] and information on the surviving cell fraction at 2 Gy (SF2) was retrieved [29]. Radiosensitive and radioresistant cancer cell lines were defined by a SF2 < 0.2 and > 0.8, respectively, as previously described [30]. Two or three replicates were available for each cell line. The second and third sets included 32 and 5 HNSCC cell lines. Raw data were downloaded from GSE79368 [23] and GSE21644 [9], respectively. In these two sets, intrinsic radioresistance was assessed by a 96-well plate clonogenic assay and the cutoff was set at a median area under the curve of 2.0 in order to identify radioresistant versus radiosensitive cell lines.

Finally, a set of 659 established cancer cell lines from 13 different cancer tissue types (nervous system, soft tissue, digestive system, bone, lung, urogenital system, aero-digestive tract, skin, kidney, breast, pancreas, blood and thyroid) was included in the analysis. Raw data was downloaded from ArrayExpress (E-MTAB-3610) [31]. The corresponding half maximal inhibitory concentration (IC_50_) for 140 drugs was retrieved from the Genomics of Drug Sensitivity in Cancer data portal [32].

Detailed information (type and number of samples, platform, and normalization) is provided in Additional file 1: Table S1.

### Primary HNSCC datasets

For the ‘The Cancer Genome Atlas’ (TCGA) dataset, RNAseqV2 normalized data (level 3, reads per kilobase per million mapped reads) of 518 primary HNSCC (421 HPV-negative and 97 HPV-positive) and 43 paired normal mucosa surrounding tumors was downloaded using the ‘TCGA2STAT’ R package [33]. From a previous publication of the comprehensive characterization of HNSCC [26], as well as from the Broad Institute TCGA GDAC Firehose using a q-value < 0.05 [34, 35], 73 genes recurrently altered at the genomic level in HNSCC were selected. Mutation status and copy number alterations from GISTIC [36] of these genes were downloaded using cBioPortal [37, 38]. Expression of 237 proteins and phosphoproteins generated by Reverse-Phase Protein Array, corresponding to 295 of the 421 primary HPV-negative HNSCC [39], was downloaded from The Cancer Proteome Atlas. For these 518 primary HNSCC, we queried the TCGA data portal as well as the cBioportal [37, 38] to retrieve clinical data, HPV status, the percentage of copy number alteration and the mutation count per sample. The molecular subtype of the 518 HNSCC was kindly provided by Vonn Andrew Walter (Lineberger Comprehensive Cancer Center, University of North Carolina at Chapel Hill, Chapel Hill, NC, USA). The percentage of tumor cells (tumor purity) was given for each tumor sample by the consensus purity estimation method and was retrieved from a previous publication [13].

Other datasets included 44 paired HNSCC and normal mucosa (GSE6631) [40], and two cohorts of 252 and 138 primary HNSCC retrieved from GSE65858 [41] and GSE39366 [25], respectively. Molecular subtypes, HPV status, and clinical information were available for tumors included in GSE39366 and GSE65858.

A detailed description of these datasets is available in Additional file 1: Table S1.

### Gene selection and computation of a RadR score

The following sequential approach was used to select genes associated with radioresistance in HPV-negative HNSCC. (1) In order to overcome the confounding effect of tumor purity [11–13], genes had to be differentially expressed in tumor versus normal mucosa; (2) among them, genes had to be differentially expressed in radioresistant versus radiosensitive HNSCC cell lines; and (3) finally, genes had to be associated with DFS in patients with HPV-negative HNSCC and treated by surgery and adjuvant radiation. Data from TCGA and GSE79368 were used as training sets for gene selection.

The single sample gene set enrichment analysis tool (ssGSEA) [42, 43] was used to compute the enrichment score (ES) using the selected genes, in the training sets (TCGA and GSE79368) and in the validation sets (GSE6631, GSE21644, NCI-60, and GSE39366). The ES of these selected genes was referred to as the RadR score. The RadR score was compared in normal versus tumor samples (GSE6631) and in radiosensitive versus radioresistant cancer cell lines (GSE21644 and NCI-60 panel), and its association with DFS was tested in GSE39366, which included patients who underwent surgical resection alone or followed by adjuvant radiotherapy alone or in combination with chemotherapy.

### Integrated analysis of the RadR score with genomic alterations, biological hallmarks, and patterns of protein expression and drug sensitivity

In the TCGA dataset, we performed an integrated analysis of the RadR score with known recurrent genomic alterations in HNSCC, genomic instability assessed by the percentage of copy number alterations (CNAs) and the mutation count provided by the cBioportal [37, 38]. Using the ssGSEA tool, we then calculated the Pearson’s coefficient of correlation of the RadR score with the ES of a collection of 48 biological hallmark gene sets previously reported [44] in three independent datasets, namely TCGA, GSE39366, and GSE65858. We then evaluated the correlation of the RadR score with the expression pattern of a panel of 237 proteins and phosphoproteins in 295 primary HPV-negative HNSCC from TCGA, as well as with expression of 188 proteins and phosphoproteins in 59 cancer cell lines from NCI-60. Finally, the RadR score was computed in 659 established cancer cell lines from various tumor types (E-MTAB-3610) and its correlation with the IC_50_ for 140 drugs was tested.

### Bioinformatics and statistical analysis

Bioinformatics and statistical analysis were performed using the Array Studio software (Omicsoft Corporation), Bioconductor packages in the R language (http://www.bioconductor.org) [45] and GraphPad Prism version 6.00 (San Diego, SA). Detailed information on processing and normalization of raw data are available in Additional file 1: Table S1. The ssGSEA tool from the GenePattern public server was used to compute the RadR score and the hallmarks’ ESs in various datasets [46]. The unpaired non-parametrical Mann–Whitney or Wilcoxon test was used to compare continuous variables between two groups and the Kruskal–Wallis test was used when more than two groups were compared. The Pearson correlation coefficient (r) was estimated to measure the strength of a linear association between two continuous variables. In the GSE39366 study, recurrence-free survival time was defined by the time in months from tumor biopsy to death, recurrence, or loss to follow-up. In the TCGA cohort, DFS was defined by the time in months from tumor biopsy to a new tumor event. Survival distributions were estimated using the Kaplan–Meier method and compared with the log‐rank test between groups of patients defined by the median RadR score (low versus high RadR score). Multivariate cox proportional hazard model, including age, node, and tumor stage, were built to select genes associated with DFS in patients treated by surgery and radiation. All statistical tests were two-sided, and *P* values of more than 0.05 were considered to be statistically significant. For multiple testing, a false-discovery rate was computed using the Bonferroni–Holm method in order to adjust *P* value.

## Results

### Identification of radioresistance-associated genes in HPV-negative HNSCC

**Fig. 1:**
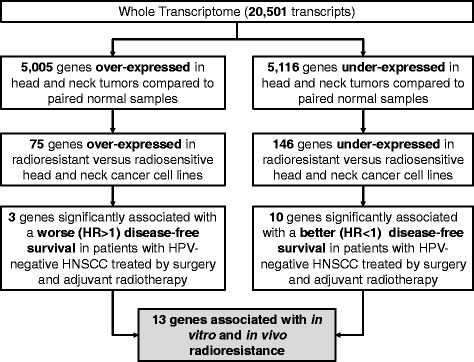
Overall strategy for the identification of 13 radioresistance-associated genes in human papillomavirus (HPV)-negative head and neck squamous cell carcinoma (HNSCC). Genes associated with HNSCC radioresistance were defined as the overlapping genes between three distinct conditions, namely genes differentially expressed in 86 paired cancer and normal mucosa samples with HNSCC from The Cancer Genome Atlas; genes differentially expressed between 14 radiosensitive and 18 radioresistant HNSCC cell lines from GSE79368; and genes associated with disease-free survival in 128 patients treated for HPV-negative HNSCC from TCGA treated with surgery and radiotherapy. We used a false discovery rate < 0.05 and a |FC| > 1 to select differentially expressed genes. *FC* fold change, *HR* hazard ratio

A sequential approach was used to select genes associated with radioresistance, by selecting (1) genes differentially expressed in tumor versus normal head and neck samples; (2) genes associated with in vitro radioresistance, and (3) genes associated with a poor DFS in patients treated by surgery and radiotherapy (Fig. 1).

In order to overcome the confounding effect of tumor purity on intrinsic radioresistance [13], we first filtered out genes that were not differentially expressed in cancer versus normal tissue using gene expression profiles of 86 paired HNSCC and normal mucosa from TCGA (FDR < 0.05, |Fold Change (FC)| > 1, paired Wilcoxon test) (Fig. 1). Among the genes differentially expressed in tumor versus normal tissue, we selected those associated with radioresistance in vitro in 32 HNSCC cell lines (18 and 14 radioresistant and radiosensitive cell lines, respectively) analyzed at baseline, 2 and 6 h after 4 Gy radiation (GSE79368) [23]. Using a two way-ANOVA independently of the time point (with a FDR < 0.05 and a |FC| > 1) (Fig. 1), all samples were included to increase the statistical power, allowing to identify 75 genes over-expressed and 146 genes under-expressed in radioresistant cell lines compared to radiosensitive ones (Fig. 1). These genes were also over- and under-expressed, respectively, in tumor compared to normal mucosa of the head and neck (Fig. 1). Among these 221 genes, we identified a set of clinically relevant genes by their association with DFS in 128 of the 421 patients with HPV-negative HNSCC from TCGA who were treated by surgery and adjuvant radiotherapy with or without concurrent chemotherapy, using a multicovariate Cox proportional hazard model, including age, node (pN0-N1 vs. pN2-N3) and tumor stage (pT1-T2 vs. pT3-T4) (Fig. 1). Clinical and pathological parameters of these 128 patients from TCGA are detailed in Additional file 2: Table S2. Median follow-up was 20.5 months. This led to the identification of 3 and 10 genes associated with increased (hazard ratio (HR) > 1, *P* < 0.05) and decreased risk (HR < 1, *P* < 0.05) of recurrence, respectively. The gene list is detailed in Table 1. Detailed information of the FC at each time points and *P* values using a Wilcoxon test for the 13 selected genes in radiosensitive and radioresistant HNSCC cell lines from GSE79368 are provided in Additional file 3: Table S3.

**Table 1:** List of 13 genes differentially expressed in tumor versus normal head and neck mucosa, associated with in vitro and in vivo radioresistance in human papillomavirus (HPV)-negative head and neck squamous cell carcinoma (HNSCC)

		Tumor vs. Normal (head neck mucosa)		Radioresistant vs. radiosensitive (HNSCC cell lines)		Association with DFS (patients with HPV-negative HNSCC treated by adjuvant radiotherapy)	
GENE		FC	FDR	FC	FDR	HR	*P* value
CSTF3	UP	1.179	0.0025	1.6031	0.0232	1.7458	0.0300
ST3GAL5	UP	1.6115	0.0083	1.9865	0.0343	1.3127	0.0495
UFD1L	UP	1.3426	<0.0001	1.45	0.0327	1.6790	0.0205
CCDC60	DN	–6.5001	<0.0001	–1.3095	0.0258	0.7688	0.0244
FAM81A	DN	–2.0136	0.0003	–1.7879	0.0473	0.7772	0.0288
FGD2	DN	–1.6559	0.0023	–1.7021	0.0464	0.7755	0.0198
HS3ST6	DN	–2.9678	0.0140	–1.3122	0.044	0.8869	0.0210
ITGB7	DN	–1.3982	0.0169	–2.5899	0.0448	0.7115	0.0050
PRR15L	DN	–6.0454	<0.0001	–2.29	0.0004	0.8618	0.0362
SCGB2A1	DN	–5.769	0.0001	–1.4528	0.0369	0.7743	0.0490
SCNN1A	DN	–4.9431	<0.0001	–4.8042	0.033	0.8432	0.0215
ST6GALNAC1	DN	–6.5319	<0.0001	–3.2498	0.0011	0.8826	0.0436
VILL	DN	–1.8517	0.0077	–2.9028	0.0055	0.7511	0.0337

*DN* downregulated, *FC* fold change, *FDR* false discovery rate, *HR* hazard ratio, *UP* upregulated

### Validation of the RadR score in HPV-negative HNSCC

**Fig. 2:**
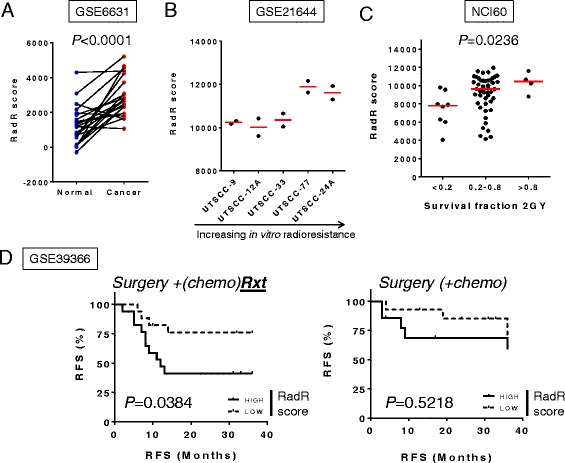
Validation of the radioresistance (RadR) score in human papillomavirus (HPV)-negative head and neck squamous cell carcinoma (HNSCC). The RadR score, defined by the gene expression-based enrichment score of 13 genes, was computed in 44 paired HNSCC and normal mucosa (GSE6631) (**a**), 5 HNSCC cell lines (GSE21644) (**b**), 59 pancancer cell lines (NCI-60) (**c**), and 63 HPV-negative HNSCC (GSE39366) (**d**). The RadR score was compared between normal and tumor samples (**a**); between HNSCC cell lines (ranked from left to right according to their increasing intrinsic radioresistance as previously defined [23]) (**b**); and between three groups of cancer cell lines from NCI60, defined by their survival fraction at 2 Gy (SF2) as previously described [30] (**c**). We divided the 63 patients from GSE39366 into two groups according to treatment as (1) surgery + adjuvant radiotherapy (with or without chemotherapy) and (2) surgery (with or without adjuvant chemotherapy). Survival distributions were estimated in each group using the Kaplan–Meier method and compared with the log‐rank test between subgroups of patients defined by the median RadR score (low versus high RadR score)

Using the ssGSEA tool, the 13-gene expression-based RadR score was computed in multiple independent datasets available from public repositories, namely GSE6631, GSE21644, GSE3966, and the NCI-60 panel. The RadR score was increased in 19/22 HNSCC tumors when compared to paired normal mucosa in GSE6631. Overall, the score was significantly increased when tumor and paired normal mucosa were compared (Wilcoxon paired test *P* < 0.0001) (Fig. 2a). Moreover, the RadR score was higher in radioresistant cell lines (UTSCC-77, UTSCC-24A) compared to the radiosensitive UTSCC-9 cell line (GSE21644) (Fig. 2b). Of note, among the five HNSCC cell lines that were profiled in duplicate by independent investigators (GSE21644), four were overlapping with the cell lines used in the training set (GSE79368). Thus, the RadR score was also computed in a larger panel of 59 cancer cell lines from various tumor types (NCI-60), which were profiled in two or three replicates (174 samples). Interestingly, the RadR score computed in each replicate was highly correlated to other replicates for the same cell line (r ≥ 0.9, *P* < 0.0001, see Additional file 4: Table S4). Using the first replicate, the RadR score was significantly different between the three groups of NCI-60 cell lines as defined by different ranges of survival fraction at 2 Gy (SF2) as shown in Fig. 2c (*P* = 0.0236, Kruskal–Wallis test). Radioresistant lines (SF2 > 0.8) had higher RadR scores compared to radiosensitive lines (SF2 < 0.2) (*P* = 0.0162, Mann–Whitney test).

The clinical relevance of the RadR score was assessed in a set of 63 primary HNSCC using GSE39366. In this study, HPV-positive tumors were excluded and patients were divided into two groups according to treatment. A high RadR score (i.e., greater than the median score) was significantly associated with a shorter recurrence-free survival (log-rank *P* = 0.0384) in 34 patients treated with surgery and adjuvant radiotherapy with or without concurrent chemotherapy (Fig. 2d). Interestingly, no difference was observed in 29 patients treated with surgery alone or with chemotherapy (*P* = 0.5218). This finding underlines the specificity of the RadR score and validates our strategy to include the selection of genes associated with in vitro radioresistance and poor DFS in patients surgically resected and treated with postoperative radiation therapy. When validated in independent cohorts, the RadR score may therefore help the clinician to tailor radiation dose according to the radioresistance level of a tumor.

In summary, these results validate the association of the RadR score with in vitro radioresistance and with the benefit from radiotherapy in patients treated with surgery and adjuvant radiotherapy but not in patients treated with surgery alone.

### The RadR score is associated with the molecular classification of HNSCC

In large genomic profiling studies of HNSCC, four distinct molecular subtypes have been consistently reported, namely atypical, basal, classical, and mesenchymal [25, 26]. HPV-positive HNSCC, which arise from the oropharynx and often fall into the “atypical” class, are recognized to be more radiosensitive compared to HPV-negative tumors [14, 15]. In line with these reports, HPV-positive HNSCC showed lower RadR scores compared to HPV-negative tumors (*P* < 0.01) in three independent sets of HNSCC (TCGA, GSE65858 and GSE39366) (Additional file 5: Figure S1).

**Fig. 3:**
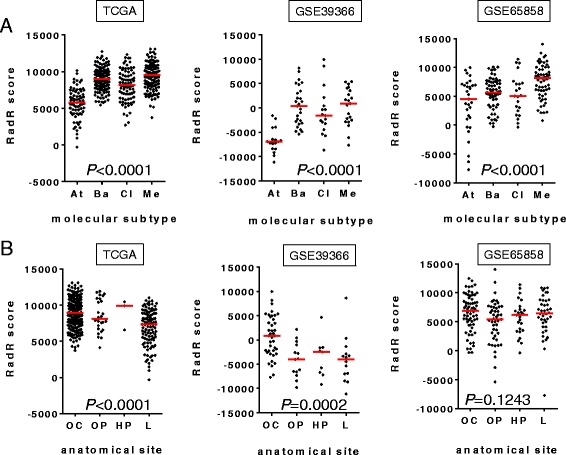
Association of the radioresistance (RadR) score with the molecular subtypes or anatomical sites of human papillomavirus (HPV)-negative head and neck squamous cell carcinoma (HNSCC). The RadR score was computed in 421 (TCGA), 179 (GSE39366), and 82 (GSE65858) primary HPV-negative HNSCC, respectively. In all three datasets, the RadR score was compared between the four molecular subtypes previously described [25, 26] (*At* atypical, *Ba* basal, *Cl* classical, *Me* mesenchymal) (**a**) as well as between four distinct anatomical sites (*OC* oral cavity, *OP* oropharynx, *HP* hypopharynx, *L* larynx) (**b**), using a Kruskal–Wallis test. *P* value is shown

We then focused on the RadR score distribution according to the molecular subtypes and the anatomical subsites in HPV-negative HNSCCs from these three independent datasets (TCGA, n = 421; GSE65858, n = 179; GSE39366, n = 82). The RadR score was found to be statistically different across molecular subtypes, and consistently lower in atypical compared to classical, basal, and mesenchymal HPV-negative HNSCC (*P* < 0.0001 in all three datasets) (Fig. 3a). Furthermore, the RadR score was different according to the anatomical subsite with a trend to be higher in the oral cavity in the TCGA (*P* < 0.0001) and GSE39366 (*P* = 0.0002) datasets, although no difference was observed in GSE65858 (*P* = 0.1243) (Fig. 3b).

Overall, our results provide evidence that, among HPV-negative HNSCC, “atypical” HNSCCs may be more sensitive to radiotherapy when compared to other subtypes, whereas “mesenchymal” HNSCCs may be more radioresistant. In contrast, the association of the anatomical subsite with the RadR score was not consistent across the three sets of data analyzed.

### Molecular subtypes of HPV-negative HNSCC, recurrent genomic alterations and RadR score

Since gene expression-based molecular subtypes have been shown to be enriched with some genomic alterations, such as *EGFR* amplification and *HRAS-CASP8* co-mutations in the classical and basal subtypes, respectively [25, 26], we then tested the association of the RadR score with genomic instability and known recurrent genomics alterations in HNSCC.

**Fig. 4:**
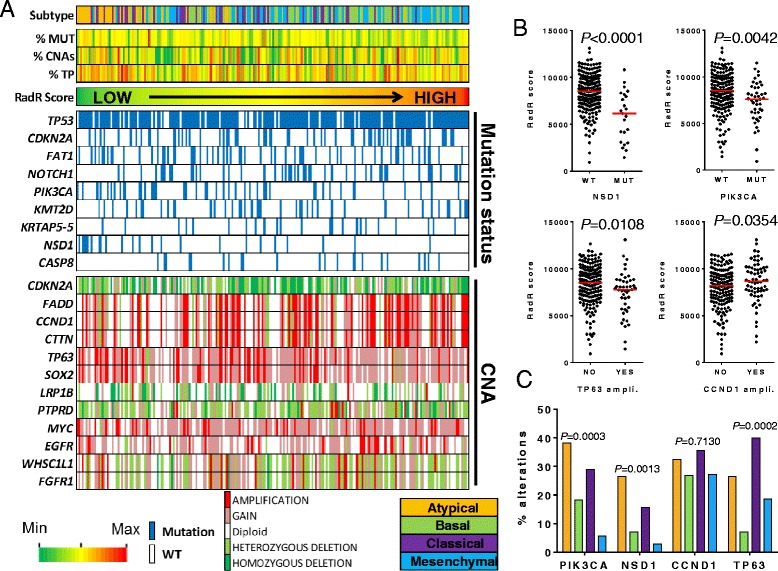
Integrative analysis of the radioresistance score with known recurrent genomic alterations in 220 human papillomavirus (HPV)-negative head and neck squamous cell carcinoma (HNSCC) from The Cancer Genome Atlas (TCGA). **a** Patients are represented by columns while specific variables are represented by rows. Tumors are ranked by the radioresistance (RadR) score computed in each tumor sample. The molecular class, the percentage of copy number alterations (%CNAs), the mutation count (%MUT), and the tumor purity (%TP) are shown for each patient. We selected known recurrently altered genes at the genomic level according to the previous publication by the TCGA network [26]. CNAs from GISTIC as well as somatic mutations of recurrently altered genes with a frequency > 10% are shown. A color range from green to red was used to represent increasing numerical values. **b** Using a Mann–Whitney test, the RadR score was compared between samples with *NSD1* and *PIK3CA* mutations compared to wild type samples, as well as between samples which harbored *CCND1* and *TP63* amplifications and those which did not. **c** Percentage of genomic alterations of *NSD1*, *PIK3CA* (mutations), and *CCND1* and *TP63* (amplifications) were compared across molecular subtypes of HPV-negative of HNSCC

No clear association was observed between the RadR score and genomic instability assessed by the percent of CNAs (r = 0.012; *P* = 0.803) and the mutation count (r = –0.204, *P* = 0.002). We then further investigated the association between the RadR score with somatic mutations and CNAs of 73 genes recurrently altered in HNSCC as reported previously [26], and defined by the Broad Institute TCGA GDAC Firehose [34, 35]. Somatic mutations as well as putative CNAs (GISTIC) of these genes were retrieved from cBioPortal and used to perform an integrated analysis of RadR score with overall genomic instability, mutation status and CNAs in 220 HPV-negative HNSCC (Fig. 4a). A decreased RadR score was significantly associated with *NSD1* (*P* < 0.0001) and *PIK3CA* (*P* = 0.0042) mutations, and to a lesser degree with *TP63* amplification (*P* = 0.0108); while HNSCC harboring *CCND1* amplification had a higher RadR score (*P* = 0.0354) (Fig. 4b). Noticeably, percentages of *NSD1* and *PIK3CA* mutations as well as *TP63* amplification were significantly higher in the atypical and classical subtypes between HPV-negative HNSCC (*P* = 0.0013, *P* = 0.0003, and *P* = 0.0002, respectively), whereas the percentage of *CCND1* amplification was not different between the four subtypes (*P* = 0.7130) (Fig. 4c).

The influence of tumor purity on genomic analysis has been well established [11–13]. We thus evaluated the association between the RadR score and the tumor purity given by the consensus purity estimation method [13]. No significant association was found in the 421 HPV-negative HNSCC from TCGA (r = –0.088, *P* = 0.0707) (Additional file 6: Figure S2), suggesting that the RadR score is independent of the tumor purity.

In summary, the RadR score was associated with some specific known recurrent genomic alterations that are known to be present at different rates in the molecular subtypes of HPV-negative HNSCC, suggesting that genomic-driven radioresistance may be different among different molecular subtypes. The lack of correlation between the RadR score and the tumor purity greatly enhances our confidence in this result.

### Molecular subtypes of HPV-negative HNSCC, biological hallmark, patterns of protein expression and the RadR score

Since few actionable associations were observed with known recurrent genomic alterations in HNSCC, we seek to gain more insights into the biological context associated with radioresistance, using an in silico functional approach based on the integrative analysis of biological pathways and proteomics data provided by The Cancer Proteome Atlas [39].

**Fig. 5:**
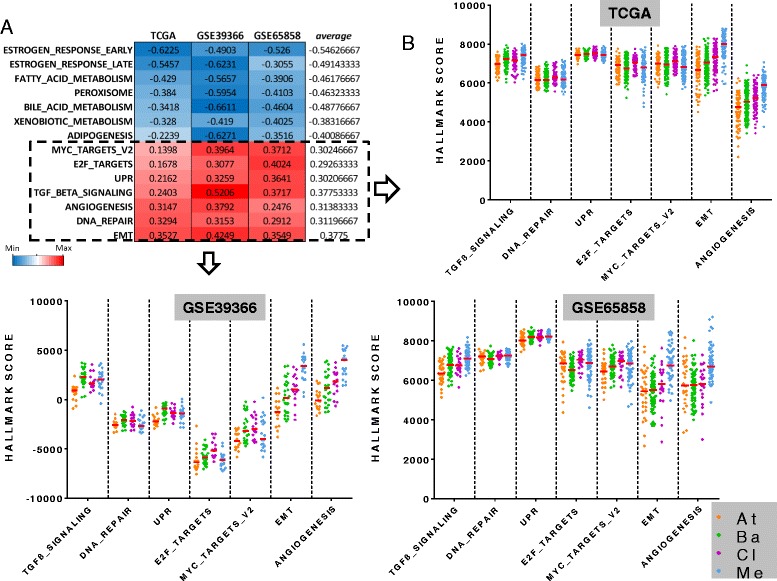
Correlation of biological hallmarks with the radioresistance (RadR) score and their distribution with molecular subtypes in human papillomavirus (HPV)-negative head and neck squamous cell carcinoma (HNSCC). **a** The RadR score was correlated to the enrichment scores (ESs) of 48 hallmarks gene sets in 421, 179, and 82 HPV-negative HNSCC from TCGA, GSE65858, and GSE39366, respectively. The 14 hallmarks significantly (*P* < 0.05) correlated in all three datasets are shown. **b** ESs of hallmarks which were positively correlated to the RadR score were compared between the four molecular subtypes of HNSCC in the three datasets. At: atypical; Ba: basal; Cl: classical; Me: mesenchymal

We computed the Pearson’s correlation of the RadR score with the ESs of 48 gene sets characterizing biological hallmarks in three independent datasets of primary HPV-negative HNSCC (TCGA, n = 421; GSE65858, n = 179; GSE39366, n = 82). In this analysis, seven hallmarks were significantly correlated with the RadR score in the three datasets (*P* < 0.01, Fig. 5a): “MYC_targets v2” (average r = 0.30), “E2F targets” (average r = 0.29), “unfolded protein response” (UPR gene set; average r = 0.30), “TGFb signaling” (average r = 0.38), “angiogenesis” (average r = 0.31), “DNA repair” (average r = 0.31), and EMT (average r = 0.38) (Fig. 5a). The ESs of these seven radioresistance-associated hallmarks were significantly different between the molecular subtypes (Kruskal–Wallis test, FDR < 0.0001). Interestingly, the mesenchymal subtype had higher ES for the hallmarks “EMT” and “angiogenesis”, whereas the classical subtype was associated with a higher ES for the hallmark “E2F targets” and “MYC targets”. Notably, the ES of the “UPR” hallmark were lower in the atypical tumors compared to others.

We then analyzed the expression of 237 proteins and phosphoproteins in 295 HPV-negative HNSCC from TCGA and tested their correlation with the RadR score. Interestingly, the most positively correlated proteins were fibronectin and PAI1 (r = 0.3486, *P* < 0.0001; r = 0.3507, *P* < 0.0001, respectively), while E-cadherin was negatively correlated (r = –0.3016, *P* < 0.0001), consistent with the association of the RadR score with EMT. Using a coefficient threshold of 0.3, we found no relevant correlation of phosphoproteins with the RadR score.

**Fig. 6:**
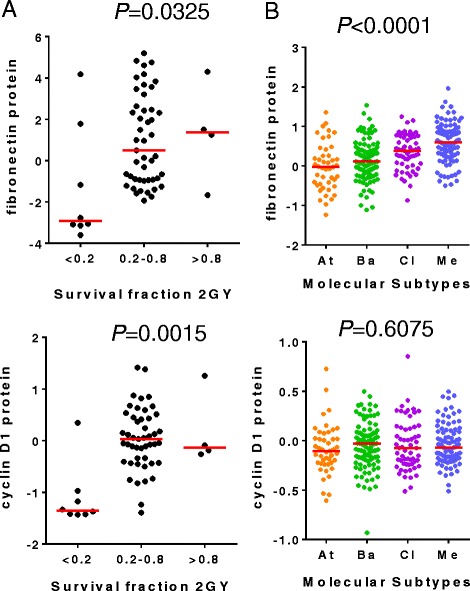
Fibronectin and cyclin D1 expression in radiosensitive and radioresistant cancer cell lines and in molecular subtypes of human papillomavirus (HPV)-negative head and neck squamous cell carcinoma (HNSCC). Protein expression levels of fibronectin and cyclin D1 were (**a**) compared between groups of NCI60 cancer cell lines defined by different ranges of survival fraction at 2 Gy (SF2) (Kruskal–Wallis test) and (**b**) compared between molecular subtypes of HNSCC in HPV-negative HNSCC from The Cancer Genome Atlas

In order to assess the association of proteins and phospho-proteins with in vitro radioresistance, we looked for proteins that were correlated to the RadR score as well as to SF2 in 59 cancer cell lines from NCI60 for consistency, using a Pearson’s correlation. A significant correlation was found for Akt (vs. RadR score: r = 0.5351, *P* < 0.0001; vs. SF2: r = 0.2836, *P* = 0.0295), fibronectin (vs. RadR score: r = 0.3687, *P* = 0.0041; vs. SF2: r = 0.259, *P* = 0.0476), cyclin D1 (vs. RadR score: r = 0.3587, *P* = 0.0053; vs. SF2: r = 0.4027, *P* = 0.0016), Irs1 (vs. RadR score: r = 0.2921, *P* = 0.0248; vs. SF2: r = 0.2885, *P* = 0.0267), Rictor pT1135 (vs. RadR score: r = 0.2742, *P* = 0.0356; vs. SF2: r = 0.3047, *P* = 0.0189), and Paxillin (vs. RadR score: r = 0.2641, *P* = 0.0433; vs. SF2: r = 0.452, *P* = 0.0003). Of note, cyclin D1 and fibronectin protein levels were increased according to the range of SF2 in cancer cell lines from NCI-60 (*P* = 0.0015 and *P* = 0.0325, respectively) (Fig. 6a). Moreover, fibronectin was significantly higher in the mesenchymal subtype of HPV-negative HNSCC, whereas no difference was observed for cyclin D1 protein expression between the four subtypes.

The association of specific biological hallmarks and proteomic alterations with the RadR score and molecular subtypes of HPV-negative HNSCC suggests that drugs targeting these hallmarks may be relevant to treat tumors with a high RadR score, according to their molecular classification. We computed the RadR score in a large panel of 659 cancer cell lines with available IC_50_ (half maximal inhibitory concentration) for 140 drugs [32]. By correlating the RadR score with the IC_50_, four drugs were positively correlated to the RadR score (r > 0.2; *P* < 0.01) (Additional file 4: Figure S3). They included cisplatin, a potent radiosensitizer routinely used in patients concurrently with radiation therapy, the pro-apoptotic Bcl-2 inhibitor TW37, the BRAF inhibitor PLX4720, and elesclomol, an inhibitor of HSP90 that modulates the UPR pathway [47, 48]. Of note, the RadR score was significantly higher in cell lines sensitive to elesclomol compared to resistant cell lines as previously defined [31] (Additional file 7: Figure S3), suggesting that targeting the radioresistance-associated UPR hallmark may be relevant in patients with a high RadR score.

Our results evidence an important role of seven specific hallmarks into the radioresistance of HPV-negative HNSCC, particularly the EMT hallmark, which is in line with an increased radioresistance observed in the mesenchymal subtype, and the UPR hallmark, which may be modulated by elesclomol in HPV-negative HNSCC with a high RadR score. The association of these hallmarks with molecular subtypes of HPV-negative HNSCC may lead to the generation of hypotheses to refine combination therapies of radiation with modulators of EMT-driven cancer cell plasticity or of the UPR pathway.

## Discussion

Radiotherapy is indicated and may benefit approximately 75% of patients with HNSCC [49], demonstrating its key role in the management of these patients. However, radiotherapy is challenging in part due to the complex anatomy of the upper aerodigestive tract, which makes it difficult to safely deliver an efficient dose to the tumor and lymph nodes when indicated. It is now well established that HPV-positive tumors are more radiosensitive [14, 15]. In this study, we focus on HPV-negative HNSCC and identify a 13-gene expression-based RadR score predictive of the benefit of radiotherapy in vitro and in patients. We show that molecular subtypes of HPV-negative HNSCC were characterized by different levels of radioresistance, where the atypical subtype was the most radiosensitive, whereas the mesenchymal was the most radioresistant, as defined by the RadR score in this population of patients. Our results suggest that the molecular classification of HPV-negative HNSCC and the RadR score may help refine radiation therapy strategies.

Gene-wide expression profiles have been previously used to identify various gene expression signatures or biomarkers associated with radiosensitivity and/or locoregional recurrence in head and neck cancers [7–10, 50]. However, the score of these signatures and biomarkers are poorly described in normal mucosa surrounding the tumor or tumor-associated normal cells, introducing bias for their interpretation in tumors with heterogeneous purity. Indeed, the influence of tumor purity on genomic analysis is well established [11–13]. In order to overcome this potential bias, we filtered out genes that were not differentially expressed between normal and tumor samples and showed that the RadR score was significantly higher in tumors compared to normal samples and was not correlated to tumor purity in the HNSCC TCGA dataset. Then, we selected genes that were differentially expressed between radioresistant and radiosensitive head and neck cancer cell lines and those which were associated with DFS in HPV-negative HNSCC from TCGA. However, because clinical information in TCGA has not been collected prospectively and monitored as in a clinical trial, the association of the RadR score with DFS needs to be validated in prospective cohorts.

Previous studies have shown a higher radiosensitivity in HPV-positive oropharyngeal SC, leading to de-escalation treatment protocols to decrease morbidity [14, 15, 17]. Consistently, the RadR scores were found to be lower in HPV-positive versus HPV-negative tumors. Moreover, the vast majority of HPV-positive tumors fall into the “atypical” molecular class in the most recent and extensive classification of HNSCC by the TCGA [26]. Intriguingly, HPV-negative tumors falling into the “atypical” molecular class had lower RadR scores in three independent datasets and may therefore be more radiosensitive and benefit from similar de-escalation strategies.

Since molecular subtypes of HNSCC exhibit different canonical genomic alterations, such as *EGFR* amplification or *HRAS* mutation [25, 26], it was tempting to hypothesize that the association of radioresistance, as defined by the RadR score, with the molecular classification may be related to specific genomic alterations. Interestingly, the *PIK3CA* mutations, most commonly found in radiosensitive HPV-positive HNSCC [26], were also more commonly found in the atypical subtype, a rather radiosensitive subtype based on low RadR scores. These observations suggest that similar molecular alterations, such as *PIK3CA* mutations, may enhance radiosensitivity in atypical tumors irrespective of their HPV status. Similarly, *NSD1* mutation and amplification of *TP63* were associated with a low RadR score and more frequent in the atypical and classical subtypes.

In order to gain more insight into the biological characterization of radioresistance in HPV-negative HNSCC and because few targetable genomic alterations were positively associated with the RadR score, we correlated the RadR score with the ES of 48 distinct biological hallmarks as well as with the expression of 237 phosphoproteins and proteins. We found seven biological hallmarks that were consistently correlated to the RadR score in the three independent datasets, namely “TGFβ signaling”, “DNA repair”, “angiogenesis”, “UPR”, “E2F targets”, “MYC targets”, and “EMT”. In line with our results, angiogenesis and DNA repair pathways are known to play an important role in radioresistance in HNSCC [22]. Moreover, the “EMT” hallmark was significantly and positively correlated to the RadR score, supporting the previously reported association of EMT with radioresistance in HNSCC [9, 23]. As expected, the mesenchymal subtype had higher ESs for the hallmarks “EMT”, “angiogenesis” and “TGFβ signaling”, as compared to other subtypes; whereas the classical subtype was characterized by higher ESs for the hallmarks “E2F targets” and “MYC targets”, thus suggesting that radioresistance may be driven by different hallmarks depending on the molecular subtype of a specific HPV-negative HNSCC.

Furthermore, we found that the RadR score was associated with patterns of drug sensitivity among the 140 drugs available for analysis in a large panel of established cancer cell lines [31, 32]. Computing the correlation between IC_50_ of these drugs and the RadR score, we found that four drugs may be active in cancer cell lines harboring high RadR scores, including the well-known potent radiosensitizer cisplatin. Interestingly, the IC_50_ of elesclomol, an inhibitor of the HSP90 chaperone protein which modulates the UPR pathway [47, 48, 51], was also negatively correlated to the RadR score, consistent with the likely important role of this pathway in HPV-negative HNSCC radioresistance. This will need to be validated in preclinical models.

## Conclusion

We report a 13-gene expression-based RadR score that is associated with specific molecular features of HPV-negative HNSCC. To the best of our knowledge, our study is the first report suggesting the clinical relevance of the molecular classification of HNSCC in order to refine radiation strategies. The predictive value of the RadR score needs to be validated in larger retrospective and prospective cohorts. Our work is hypothesis-generating for the refinement of combination therapies of radiation with modulators of EMT-driven cancer cell plasticity or of the UPR pathway.

## Additional files

Additional file 1: Table S1. Description of the datasets used in the study. (DOCX 14 kb)

Additional file 2: Table S2. Clinical and pathological parameters of the cohort from TCGA for selection of genes associated with disease-free survival. This cohort includes 128 patients suffering from human papillomavirus (HPV)-negative head and neck squamous cell carcinoma (HNSCC) and who were treated by surgery and adjuvant radiotherapy. (DOCX 16 kb)

Additional file 3: Table S3. Wilcoxon test between radiosensitive and radioresistant head and neck squamous cell carcinoma (HNSCC) cell lines at each time point (baseline: 0 Gy, 2 h after 4 Gy, and 6 h after 4 Gy). FC, fold change; *P*, *P* value. (DOCX 15 kb)

Additional file 4: Table S4. Correlation of the RadR score between the different replicates of each cell lines from NCI-60. (DOCX 36 kb)

Additional file 5: Figure S1. The radioresistance score in human papillomavirus (HPV)-positive compared to HPV-negative head and neck squamous cell carcinoma (HNSCC) from TCGA, GSE39366, and GSE65858. *P* value is shown for each dataset. (PPTX 189 kb)

Additional file 6: Figure S2. Correlation between tumor purity and RadR score in the 421 primaryHPV-negative HNSCC from TCGA. Pearson’s coefficient of correlation as well as P-value are shown. (PPTX 114 kb)

Additional file 7: Figure S3. RadR score and in vitro sensitivity to Elesclomol, TW37, Cisplatine andPLX4720 in >700 cancer cell lines from the Genomics of Drug Sensitivity in Cancer database. TheRadR score was compared between sensitive and resistant cell lines to Cisplatin, TW47, PLX4720 andelesclomol. Number of tested samples is shown for each drug. (PPTX 494 kb)

## Abbreviations

CNAs: copy number alterations
DFS: disease-free survival
EMT: epithelial-to-mesenchymal transition
ES: enrichment score (computed using single sample Gene Set Enrichment Analysis)
FC: fold change
FDR: false discovery rate
HNSCC: head and neck squamous cell carcinomas
HPV: human papillomavirus
HR: hazard ratio
IC_50_: half maximal inhibitory concentration (a measure of drug sensitivity)
RadR: radioresistance
SF2: survival fraction at 2 Gy (a measure of radiosensitivity)
ssGSEA: single sample gene set enrichment analysis (for computing an enrichment score of a gene set in a unique sample)
TCGA: The Cancer Genome Atlas (Public repository)
UPR: unfolded protein response
