# Supplementary material for: A 13-gene expression-based radioresistance score highlights the heterogeneity in the response to radiation therapy across HPV-negative HNSCC molecular subtypes
Source: BMC Med. 2017 Sep 1;15:165. doi: 10.1186/s12916-017-0929-y (PMC5580222; doi:10.1186/s12916-017-0929-y)
Supplement: Supplementary file 1 — Description of the datasets used in the study. (DOCX 14 kb) [file 12916_2017_929_MOESM1_ESM.docx]

**Additional Table S1: Description of the datasets used in our study**

| Datasets | Sample type | Total  (n=) | Platform | Normalization | Ref |
| --- | --- | --- | --- | --- | --- |
| NCI60 | Cancer cell lines | 174 samples  (two or three replicates)  **59** unique cell lines | Affymetrix Human Genome U133 Plus 2.0 | Raw data (CEL files) were processed using quantile normalization and the robust multi-array average (RMA) algorithm, in addition to the custom CDF version 18 from brainarray. Data was then log_2_ transformed. | [1, 2] |
| GSE79368 | HNSCC cell lines | **32** | Illumina HumanWG-6 v3.0 | Normalized data as described in the original publication were directly extracted from Gene Expression Omnibus data portal. | [3] |
| GSE21644 | HNSCC cell lines | **5** | Affymetrix Human Genome U133 Plus 2.0 | Raw data (CEL files) were processed using quantile normalization and the robust multi-array average (RMA) algorithm, in addition to the custom CDF version 18 from brainarray. Data was then log_2_ transformed. | [4] |
| E-MTAB-3610 | Sanger cell lines | 1,018  **(659** cell lines with available IC50 data) | Affymetrix Human Genome U133 Plus 2.0 | Raw data (CEL files) were processed using quantile normalization and the robust multi-array average (RMA) algorithm, in addition to the custom CDF version 18 from brainarray. Data was then log_2_ transformed. | [5] |
| GSE6631 | Paired normal and cancer HN samples | **44** | Affymetrix Human Genome U95 v2 | Raw data (CEL files) were processed using quantile normalization and the robust multi-array average (RMA) algorithm, in addition to the custom CDF version 18 from brainarray. Data was then log_2_ transformed. | [6] |
| TCGA | HNSCC  Normal mucosa | 518  (**421** HPV-negative)  **43** | Illumina HiSeq 2000 RNA sequencing | RNA seq version 2 (level III). Normalized read counts using the RPKM method were log_2_ transformed | [7] |
| GSE39366 | Primary HNSCC | 138  (**82** HPV-negative) | Agilent-UNC-custom-4X44K | Normalized data as described in the original publication were directly extracted from Gene Expression Omnibus data portal. | [8] |
| GSE65858 | Primary HNSCC | 252  (**179** HPV-negative) | Illumina HumanHT-12 V4.0 | Normalized data as described in the original publication were directly extracted from Gene Expression Omnibus data portal. | [9] |

HNSCC: Head and Neck Squamous Cell Carcinomas; IC50: half maximal inhibitory concentration

**References**

1. Reinhold WC, Sunshine M, Liu H, Varma S, Kohn KW, Morris J, Doroshow J, Pommier Y: **CellMiner: a web-based suite of genomic and pharmacologic tools to explore transcript and drug patterns in the NCI-60 cell line set**. *Cancer Res* 2012, **72**(14):3499-3511.

2. Shankavaram UT, Varma S, Kane D, Sunshine M, Chary KK, Reinhold WC, Pommier Y, Weinstein JN: **CellMiner: a relational database and query tool for the NCI-60 cancer cell lines**. *BMC Genomics* 2009, **10**:277.

3. de Jong MC, Ten Hoeve JJ, Grenman R, Wessels LF, Kerkhoven R, Te Riele H, van den Brekel MW, Verheij M, Begg AC: **Pretreatment microRNA Expression Impacting on Epithelial-to-Mesenchymal Transition Predicts Intrinsic Radiosensitivity in Head and Neck Cancer Cell Lines and Patients**. *Clin Cancer Res* 2015, **21**(24):5630-5638.

4. Jerhammar F, Ceder R, Garvin S, Grenman R, Grafstrom RC, Roberg K: **Fibronectin 1 is a potential biomarker for radioresistance in head and neck squamous cell carcinoma**. *Cancer Biol Ther* 2010, **10**(12):1244-1251.

5. Iorio F* KT, Vis DJ*, Bignell GR*, Menden MP*, Schubert M, Aben N, Gonçalves E, Barthorpe S, Lightfoot H, Greninger P, van Dyk E, Chang H, de Silva H, Heyn HA, Deng X, Egan RK, Liu Q, Mironenko T, Mitropoulos X, Richardson L, Wang J, Zhang T, Moran S, Saylos S, Soleimani M, Tamborero D, Lopez-Bigas N, Ross-Macdonald P, Esteller M, Gray N, Haber DA, Stratton MR, Benes CH, Wessels LFA+, Saez-Rodriguez J+, McDermott U+, Garnett MJ+ **A landscape of pharmacogenomic interactions in cancer** *Cell, In press* 2016.

6. Kuriakose MA, Chen WT, He ZM, Sikora AG, Zhang P, Zhang ZY, Qiu WL, Hsu DF, McMunn-Coffran C, Brown SM *et al*: **Selection and validation of differentially expressed genes in head and neck cancer**. *Cell Mol Life Sci* 2004, **61**(11):1372-1383.

7. Wan YW, Allen GI, Liu Z: **TCGA2STAT: simple TCGA data access for integrated statistical analysis in R**. *Bioinformatics* 2016, **32**(6):952-954.

8. Walter V, Yin X, Wilkerson MD, Cabanski CR, Zhao N, Du Y, Ang MK, Hayward MC, Salazar AH, Hoadley KA *et al*: **Molecular subtypes in head and neck cancer exhibit distinct patterns of chromosomal gain and loss of canonical cancer genes**. *PLoS One* 2013, **8**(2):e56823.

9. Wichmann G, Rosolowski M, Krohn K, Kreuz M, Boehm A, Reiche A, Scharrer U, Halama D, Bertolini J, Bauer U *et al*: **The role of HPV RNA transcription, immune response-related gene expression and disruptive TP53 mutations in diagnostic and prognostic profiling of head and neck cancer**. *Int J Cancer* 2015, **137**(12):2846-2857.
