# Supplementary material for: A 13-gene expression-based radioresistance score highlights the heterogeneity in the response to radiation therapy across HPV-negative HNSCC molecular subtypes
Source: BMC Med. 2017 Sep 1;15:165. doi: 10.1186/s12916-017-0929-y (PMC5580222; doi:10.1186/s12916-017-0929-y)
Supplement: Supplementary file 2 — Clinical and pathological parameters of the cohort from TCGA for selection of genes associated with disease-free survival. This cohort includes 128 patients suffering from human papillomavirus (HPV)-negative head and neck squamous cell carcinoma (HNSCC) and who were treated by surgery and adjuvant radiotherapy. (DOCX 16 kb) [file 12916_2017_929_MOESM2_ESM.docx]

**Additional Table S2: Clinical and pathological parameters of the cohort from TCGA for selection of genes associated with disease-free survival.** This cohort includes 128 patients suffering from HPV-negative HNSCC and who were treated by surgery and adjuvant radiotherapy.

| Parameter | N (%) |
| --- | --- |
| Age (years)  [min-max]  Median | [19-82]  60 |
| Gender  Male  Female | 100 (78.1)  28 (21.9) |
| Smoking habit  Never smoker  Current smoker  Reformed  NA | 28 (21.9)  52 (40.6)  47 (36.7)  1 (0.8) |
| Tumor site  Oral cavity  Oropharynx  Larynx  NA | 72 (56.3)  6 (4.7)  36 (28.1)  14 (10.9) |
| Tumor Stage  T1  T2  T3  T4 | 8 (6.3)  27 (21.1)  27 (21.1)  66 (51.6) |
| Node stage  N0  N1  N2  N3 | 44 (34.4)  21 (16.4)  62 (48.4)  1 (0.8) |
| Median follow-up (months)  All patients  Patients who recurred/progressed  Patients who did not recur/progress | 20.5  12.8  32.6 |
