# Supplementary material for: A 13-gene expression-based radioresistance score highlights the heterogeneity in the response to radiation therapy across HPV-negative HNSCC molecular subtypes
Source: BMC Med. 2017 Sep 1;15:165. doi: 10.1186/s12916-017-0929-y (PMC5580222; doi:10.1186/s12916-017-0929-y)
Supplement: Supplementary file 3 — Wilcoxon test between radiosensitive and radioresistant head and neck squamous cell carcinoma (HNSCC) cell lines at each time point (baseline: 0 Gy, 2 h after 4 Gy, and 6 h after 4 Gy). FC, fold change; P, P value. (DOCX 15 kb) [file 12916_2017_929_MOESM3_ESM.docx]

**Additional Table S3: Wilcoxon tests between radiosensitive and radioresistant HNSCC cell lines at each time point (baseline: 0Gy, two hours after 4Gy and six hours after 4 Gy).** FC: fold change; *P*: p-value

| **Gene** | | **Baseline (0 Gy)** | | **2 hours after 4Gy** | | **6 hours after 4Gy** | |
| --- | --- | --- | --- | --- | --- | --- | --- |
| **Symbol** | **ID** | **FC** | ***P*** | **FC** | ***P*** | **FC** | ***P*** |
| **CSTF3** | **1479** | 1.8693 | 0.0128 | 1.7631 | 0.0113 | 1.2501 | 0.398 |
| **ST3GAL5** | **8869** | 2.3838 | 0.0223 | 1.9774 | 0.0708 | 1.6631 | 0.1453 |
| **UFD1L** | **7353** | 1.5292 | 0.007 | 1.4714 | 0.0494 | 1.3548 | 0.2204 |
| **CCDC60** | **160777** | -1.2482 | 0.0912 | -1.5171 | 0.0031 | -1.1858 | 0.1929 |
| **FAM81A** | **145773** | -2.2311 | 0.1801 | -1.5743 | 0.7502 | -1.6272 | 0.4643 |
| **FGD2** | **221472** | -1.908 | 0.0304 | -1.6478 | 0.0542 | -1.5684 | 0.0989 |
| **HS3ST6** | **64711** | -1.2858 | 0.1251 | -1.3001 | 0.0839 | -1.3517 | 0.135 |
| **ITGB7** | **3695** | -3.1588 | 0.0371 | -2.5217 | 0.2351 | -2.1808 | 0.0912 |
| **ATAD4** | **79170** | -2.3113 | 0.0041 | -2.2655 | 0.007 | -2.2936 | 0.0061 |
| **SCGB2A1** | **4246** | -1.1718 | 0.1159 | -1.366 | 0.0006 | -1.9157 | 0.016 |
| **SCNN1A** | **6337** | -5.4328 | 0.0542 | -5.2116 | 0.0494 | -3.9162 | 0.1251 |
| **ST6GALNAC1** | **55808** | -3.2651 | 0.0839 | -3.6436 | 0.0371 | -2.8849 | 0.016 |
| **VILL** | **50853** | -3.2651 | 0.0113 | -3.2801 | 0.0089 | -2.2839 | 0.0409 |
