# Supplementary material for: A 13-gene expression-based radioresistance score highlights the heterogeneity in the response to radiation therapy across HPV-negative HNSCC molecular subtypes
Source: BMC Med. 2017 Sep 1;15:165. doi: 10.1186/s12916-017-0929-y (PMC5580222; doi:10.1186/s12916-017-0929-y)
Supplement: Supplementary file 4 — Correlation of the RadR score between the different replicates of each cell lines from NCI-60. (DOCX 36 kb) [file 12916_2017_929_MOESM4_ESM.docx]

**Additional Table S4: Correlation of the RadR score between the different replicates of each cell line from NCI-60.** Pearson’s coefficients of the correlation between technical replicates of each cell lines from three different experiments.

| **RadR score** | Replicate N°1 | Replicate N°2 | Replicate N°3 |
| --- | --- | --- | --- |
| Replicate N°1 | 1 | 0.944 | 0.900 |
| Replicate N°2 | 0.944 | 1 | 0.904 |
| Replicate N°3 | 0.900 | 0.904 | 1 |
