# Supplementary figures and images for: A 13-gene expression-based radioresistance score highlights the heterogeneity in the response to radiation therapy across HPV-negative HNSCC molecular subtypes
Source: BMC Med. 2017 Sep 1;15:165. doi: 10.1186/s12916-017-0929-y (PMC5580222; doi:10.1186/s12916-017-0929-y)

## Slide 1
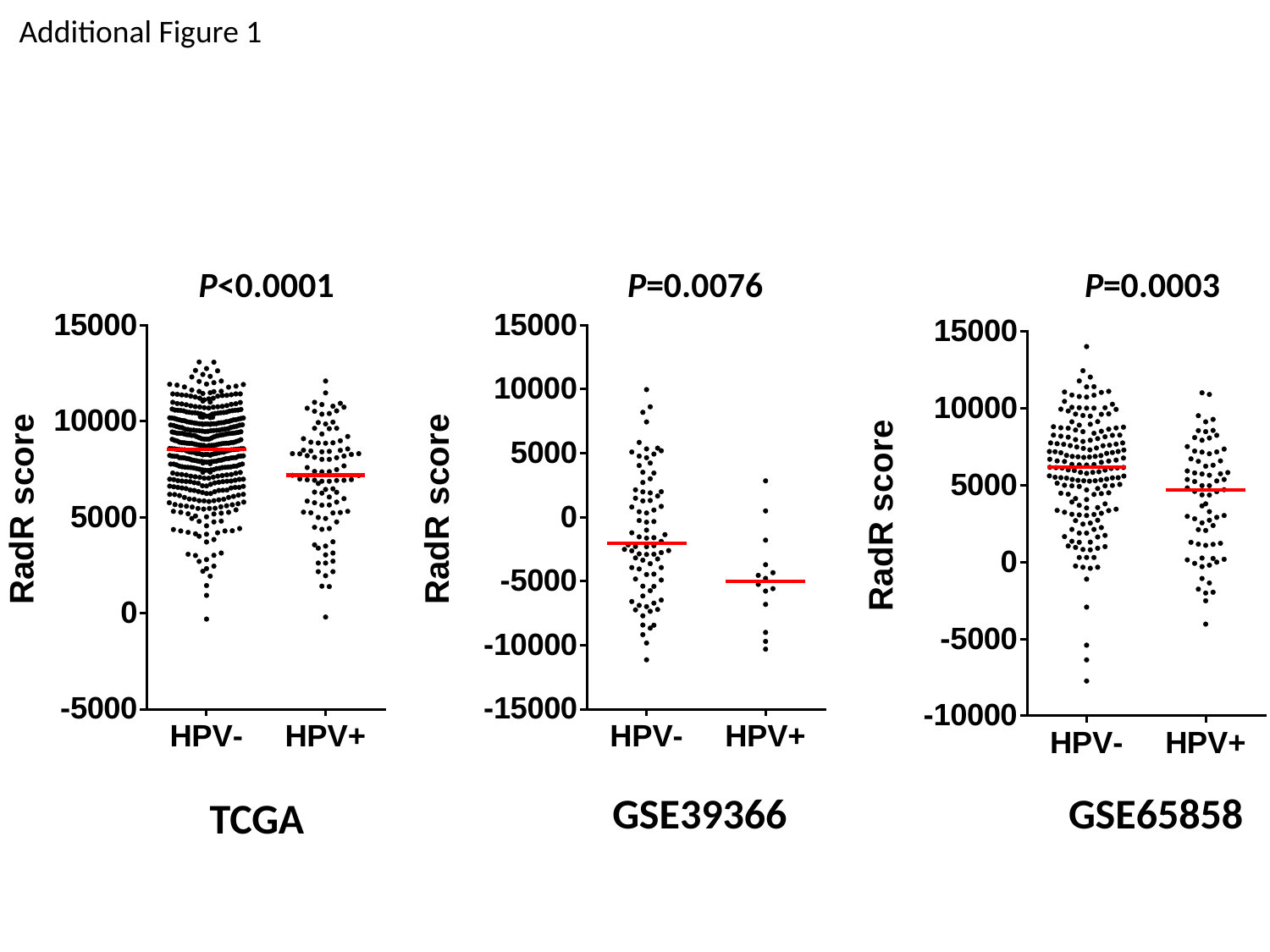

Additional Figure 1
P<0.0001
P=0.0076
P=0.0003
GSE39366
GSE65858
TCGA

Supplement: Supplementary file 5 — The radioresistance score in human papillomavirus (HPV)-positive compared to HPV-negative head and neck squamous cell carcinoma (HNSCC) from TCGA, GSE39366, and GSE65858. P value is shown for each dataset. (PPTX 189 kb) [file 12916_2017_929_MOESM5_ESM.pptx]

## Slide 1
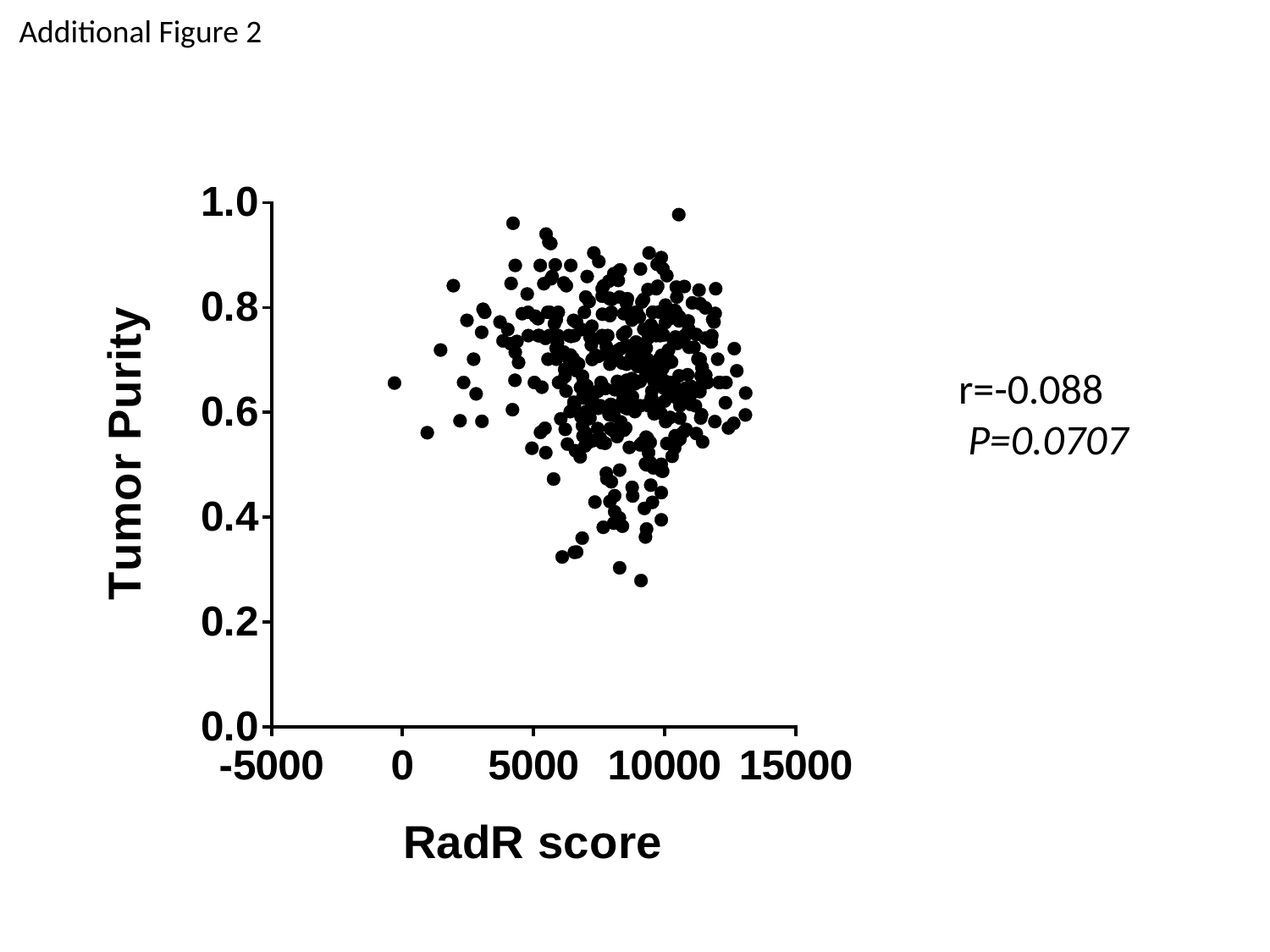

Additional Figure 2
r=-0.088
 P=0.0707

Supplement: Supplementary file 6 — Correlation between tumor purity and RadR score in the 421 primaryHPV-negative HNSCC from TCGA. Pearson’s coefficient of correlation as well as P-value are shown. (PPTX 114 kb) [file 12916_2017_929_MOESM6_ESM.pptx]

## Slide 1
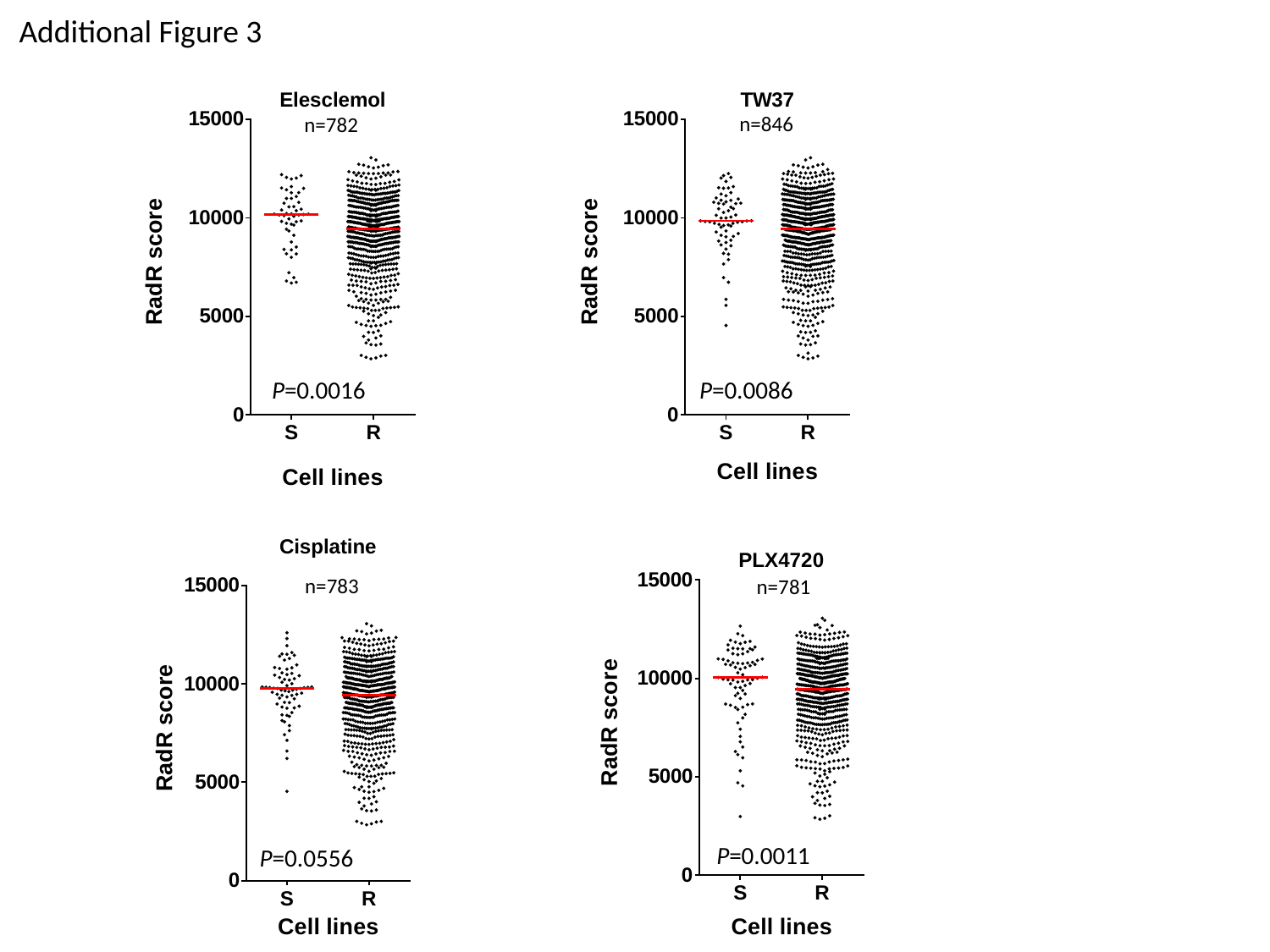

Additional Figure 3
n=846
n=782
P=0.0086
P=0.0016
n=783
n=781
P=0.0011
P=0.0556

Supplement: Supplementary file 7 — RadR score and in vitro sensitivity to Elesclomol, TW37, Cisplatine andPLX4720 in >700 cancer cell lines from the Genomics of Drug Sensitivity in Cancer database. TheRadR score was compared between sensitive and resistant cell lines to Cisplatin, TW47, PLX4720 andelesclomol. Number of tested samples is shown for each drug. (PPTX 494 kb) [file 12916_2017_929_MOESM7_ESM.pptx]
